# Supplementary material for: Phase separation by the polyhomeotic sterile alpha motif compartmentalizes Polycomb Group proteins and enhances their activity
Source: Nat Commun. 2020 Nov 5;11:5609. doi: 10.1038/s41467-020-19435-z (PMC7644731; doi:10.1038/s41467-020-19435-z)
Supplement: Supplementary file 1 — Supplementary Information [file 41467_2020_19435_MOESM1_ESM.pdf]

Supplementary Figure 1 Proteins and Chromatin used in these experiments. A. SYPRO Ruby stained SDS-PAGE of Mini-Ph and its derivatives that were prepared in *E. coli*. Equimolar amounts of each protein were loaded. B. Ruby stained gel of 3-subunit PRC1 consisting of Flag-PSC, Pc, and dRING (PRC1ΔPh). C. Schematic of plasmid used for chromatin assembly. The plasmid used for most experiments consists of 40*5S nucleosome positioning sequences (208 base pair repeat) (blue), and the plasmid backbone (green). Each 5S sequence is flanked by EcoRI sites. To estimate nucleosome assembly, chromatinized plasmids are digested with EcoRI, and the naked and nucleosomal 5S repeats resolved by native PAGE. D. Representative gel of EcoRI digest. Left shows DNA stain used to quantify naked and nucleosomal repeats, and right side shows the Cy3 label on histone H2A. E. Micrococcal nuclease analysis of chromatin used for condensate formation. Numbers on the right indicate fragments representing nucleosomal increments. The EcoRI assay was performed for all chromatin assemblies used in this work, and micrococcal nuclease for at least 4 independent chromatin preparations.

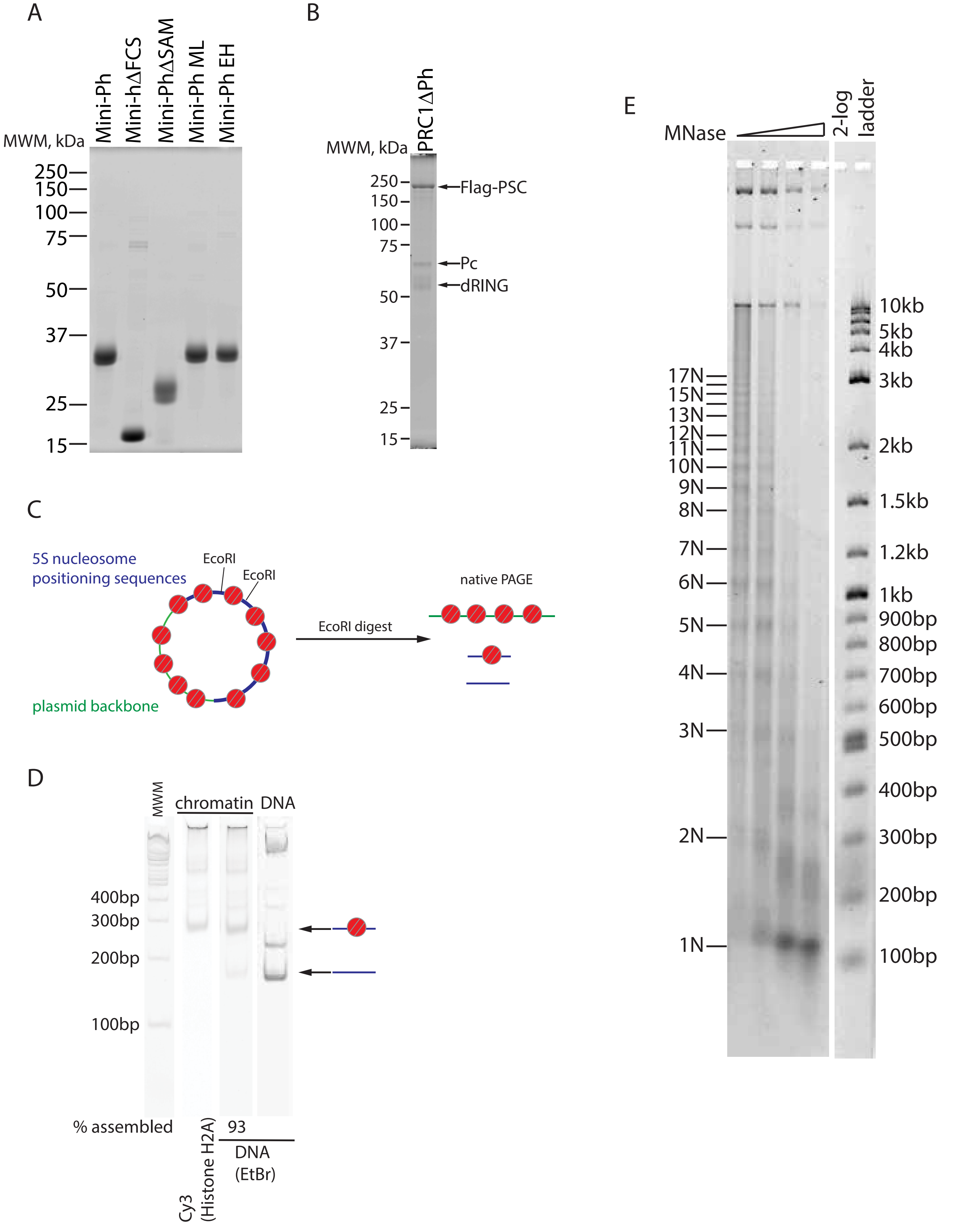

Seif et al., Supplementary Figure 1

Supplementary Figure 2 Mini-Ph forms phase separated condensates with DNA. A. Mini-Ph forms phase separated condensates with DNA. B. Time-lapse of droplet fusion of Mini-Ph-chromatin condensates, visualized with Alexa 647-labelled Mini-Ph. These observations were consistent with three different preparations of Mini-Ph. C-E. Mini-Ph-DNA condensates can be pelleted by brief centrifugation. Representative gels (C) of DNA (left) and protein (fluorescent scan of Alexa 647-labelled Mini-Ph). Condensates were allowed to form for 15 min. at room temperature. Reactions are as follows: 1 DNA; 2 Mini-Ph; 3 DNA + Mini-Ph. D. Quantification of pelleting experiments. p-values are for two-tailed paired student's t-test. E. Mini-Ph-DNA condensates increase turbidity measured by OD_{340}. p-values are for one-way ANOVA with paired samples and Sidak correction for multiple comparisons. For D and E, n=3 independent experiments. F. Matrix of Mini-Ph and DNA. Representative of two independent experiments. G. Graph of one-phase versus two-phase regions of the Mini-Ph-DNA matrix. Note that high concentrations of DNA disrupt condensate formation (e.g. 46 nM DNA with 5 μM Mini-Ph). [DNA] refers to the concentration of plasmids.

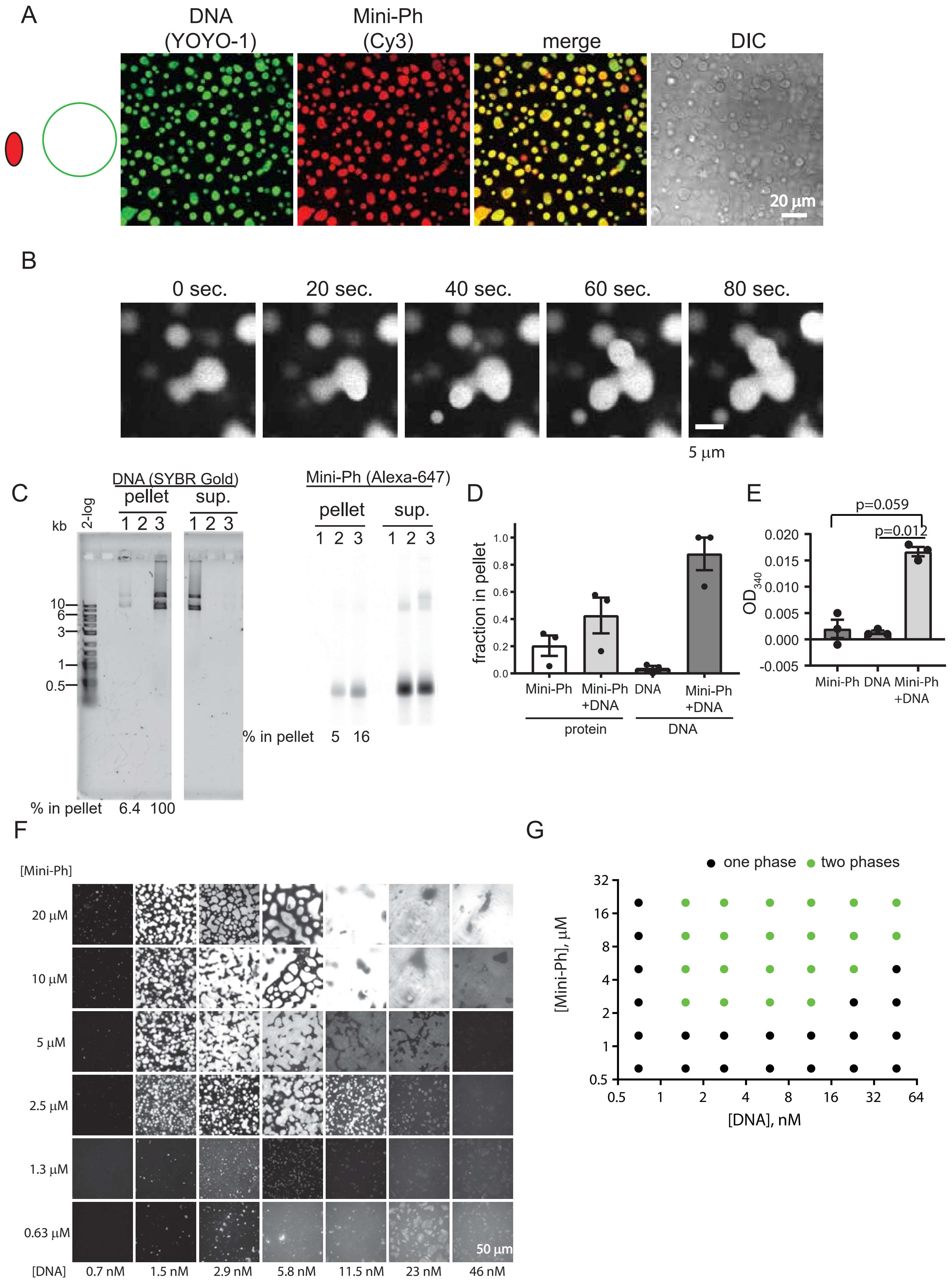

Seif et al., Supplementary Figure 2

Supplementary Figure 3 Mini-Ph-DNA condensates are sensitive to NaCl concentration. A, B. Matrix of of Mini-Ph-DNA condensates and across different concentrations of NaCl. A shows representative images, and B the graph of one-phase versus two-phase points. C. Increasing [NaCl] disrupts condensates as measured by OD_{340}. Mean +/- SD of three titrations.

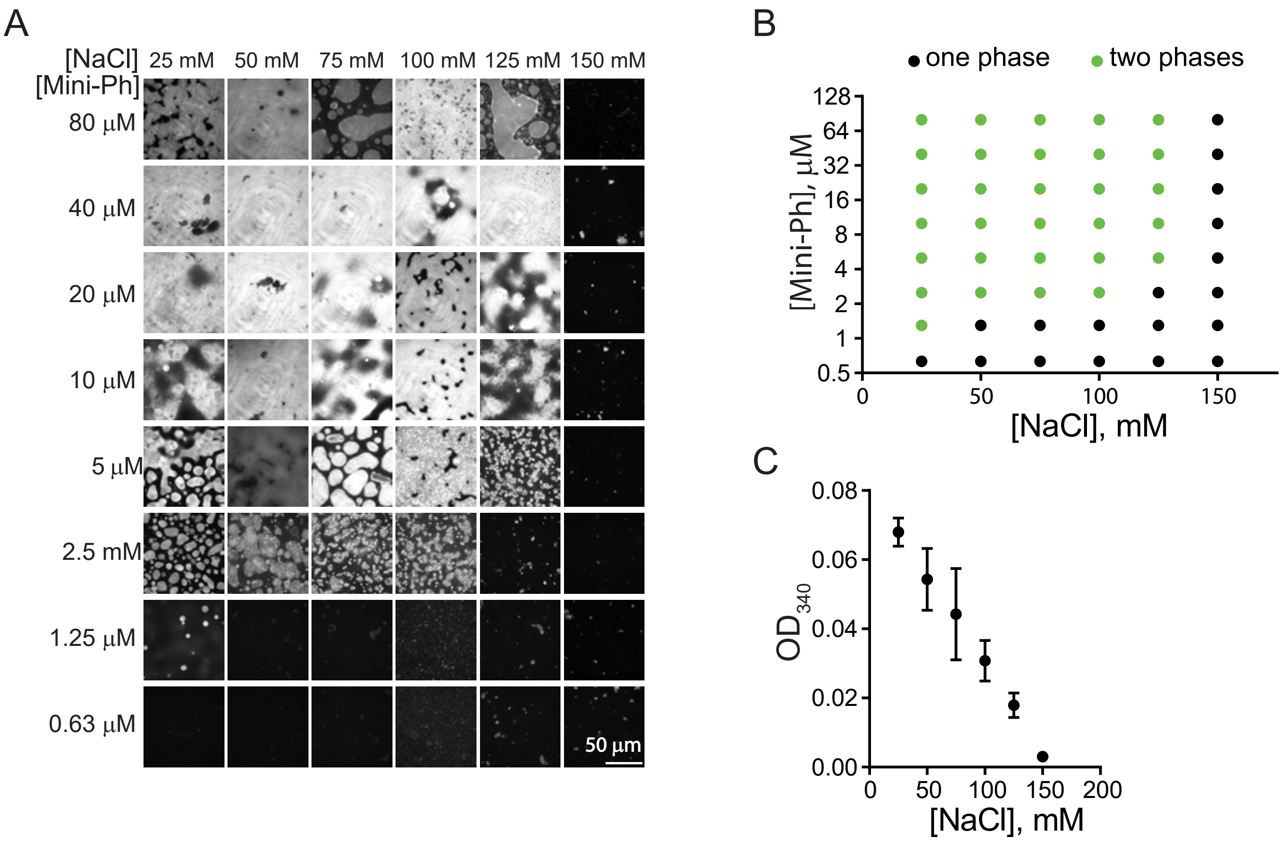

Seif et al., Supplementary Figure 3

Supplementary Figure 4 Sequence properties of Mini Ph linker. A. Colour coded Mini-Ph linker sequence. Gray and red lines indicate linker residues that may interact with Ph SAM based on NMR results with the linker and the SAM in trans. Red line indicates residues that had altered NMR signals when mixed with 0.4 molar equivalents of Ph SAM, and gray lines those with altered signals with 1.6 molar equivalents of Ph SAM. See Robinson et al. (2012, JBC) for details. B. Plot of net charge per residue (NCPR, pH 8.0) of Mini-Ph linker indicating charge distribution in the linker. C. Plot of NCPR (pH 8.0) for PHC3, indicating distinct charge patterning compared with Ph-p. D. Das-Pappu diagram of states for both *Drosophila* and all three human Ph linkers, showing that the *Drosophila* and human linkers have distinct properties. E. Summary of properties of Ph linkers calculated with http://pappulab.github.io/localCIDER/. See also Supplementary Table 1 for all sequences and parameters (FCR=fraction charged residues). F. Alignment of Ph-p and Ph-d linkers. G. Alignment of PHC1-3 (human Ph homologues) linkers.

A

Mini-Ph linker

GVGSGETNGL GTGGIVGVDA MALVDRLDEA MAEEKMQTEA TPKLSESFPI
LGASTEVPPM SLPVQAAISA PSPLAMPLGS PLSVALPTLA PLSVVTSGAA
PKSSEVNGTD R

positively charged
negatively charged
aromatic
polar
aliphatic
proline

B

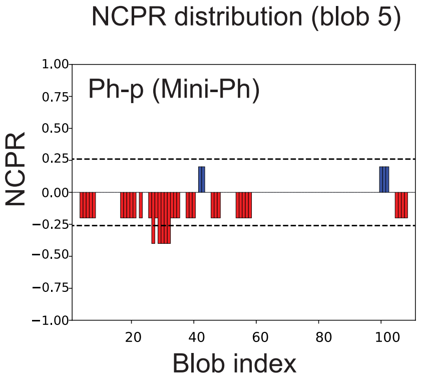

C

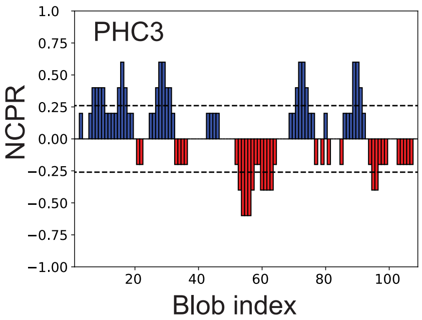

D

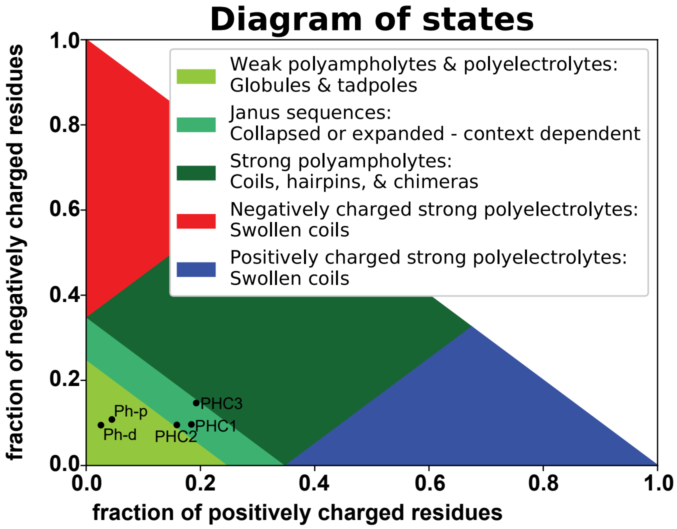

E

protein	FCR, pH 8	NCPR, pH 8	PI	fr. Neg	fr. Pos	fr. expanding, pH 8	fr. disord. pro.	kappa
Ph-p	0.153	-0.063	3.9	0.108	0.045	0.261	0.703	0.115
Ph-d	0.121	-0.069	3.6	0.095	0.026	0.233	0.647	0.128
PHC1	0.281	0.088	10.9	0.096	0.184	0.368	0.746	0.291
PHC2	0.254	0.063	10.1	0.095	0.159	0.325	0.762	0.233
PHC3	0.339	0.046	10.5	0.147	0.193	0.422	0.734	0.220

F

```
Ph-p GVGSGETNGLGTGGIVGVDAMALVDRLDEAMAEEKMQTEATPKLSESFPILGASTEVPPM
Ph-d GVGSGETNGLGTGGIVGVDAMALVDRLDEAMAEEKMQTESYQTVSDALPIQAATPEVPPI

Ph-p SLPVQAAISAPSPLAMPLGSPLSVALPTLAPLSVVTSGA-----APKSSEVNGTDR
Ph-d SMPVLAAMSTSSPLSLPLTLPLPIAIAPTVSLPVVSAGVVAPVLAIPSSNINGSDR
```

G

```
PHC1 RYNVSCSHQFRLKRKKMKEFQEANYAR-VRRRGPRRSSSDI------------ARAKIQGK
PHC2 RYNVGCTKRVGLFHSDRSKLQKAGAATHNRRRASKASLPPLTKDTKKQPTGTVPLSVTAA
PHC3 RYNVSCSKKFALSRWNRKPDNQSL-----GHRGRRPSGPD-----------GAAREHILRQ

PHC1 --CHRGQEDSSRGSDNSSYDEALSPTSPGPLSVRAGHGERDLGNPNTAPPTPELHGINPV
PHC2 LQLTHSQEDSSRCSDNSSYEEPLSPISASSSTSRRRQGQRDLELPDMH--MRDLVGMGHH
PHC3 LPITYPSAEEDLASHEDSVPSAMTTRLRRQSERERERELRDVRIRKMPE-NSDLLPVAQ-

PHC1 FLSSNPS-R
PHC2 FLPSEPT-K
PHC3 ---TEPSIW
```

Seif et al., Supplementary Figure 4

Supplementary Figure 5 Quantification of nucleosome concentration in condensates. A. Standard curve prepared from serial dilution of Cy3-labelled (on H2A) histone octamers. Buffer alone was used to collect a background intensity, which was subtracted, from all images. B. [nucleosomes] in individual structures measured from two reactions carried out with different chromatin assemblies at a starting concentration of 150 nM. Chromatin 2 was assembled at higher nucleosome density, presumably explaining the higher nucleosome concentration in condensates. n=944 and 1662 structures measured for chromatins 1 and 2. C. Example image used for quantification of nucleosome concentration. D. Outlines of thresholded structures selected for quantification for the image shown in C. E. Graph of experiment comparing concentrations of structures measured with a 1:9 mixture of Cy3-labelled (0.1X) to unlabelled (1X) chromatin versus all Cy3-labelled. The calculated concentrations are similar (21+/-4 µM, n=858 structures, and 26+/-5 µM, n=815 structures) in both reactions, suggesting the measurements in the dense phase with all labelled chromatin are in the linear range. This experiment was done a single time.

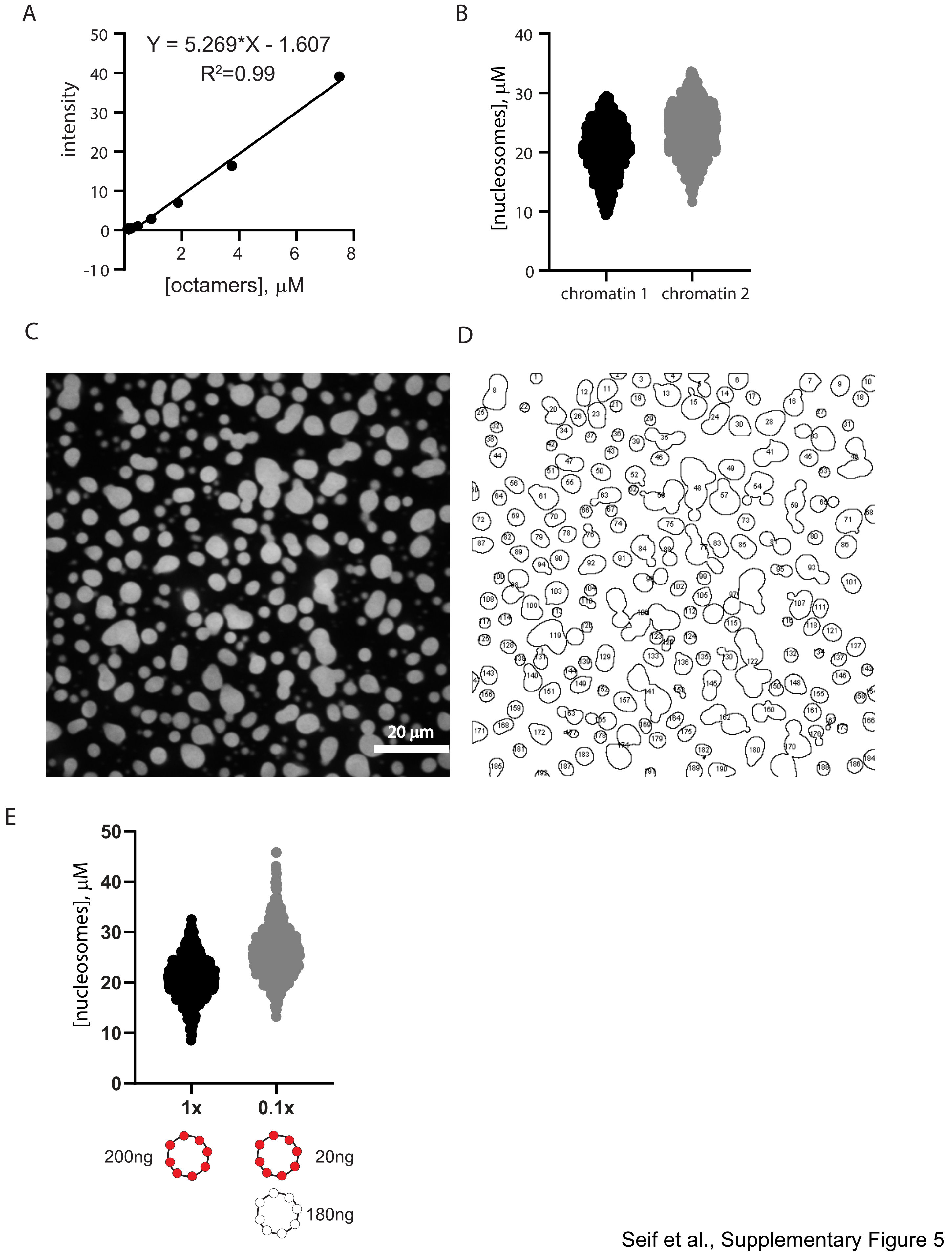

Seif et al., Supplementary Figure 5

Supplementary Figure 6 Kinetics of chromatin and Mini-Ph in condensates are distinct. A-D. Summary of parameters from double exponential fits of FRAP data. All bars show mean +/- SEM of parameters from fits (n=6 FRAP traces of single structures). Traces were obtained in two independent experiments. E. Time lapse of recovery of chromatin in a condensate. The DNA component of chromatin was visualized (with YOYO1) and bleached. F. Time lapse of recovery of chromatin in a condensate after photobleaching. H2A-Cy3 was visualized. Data are representative of results observed in two independent experiments, and in the three experiments analyzed in Supplementary Figure 14.

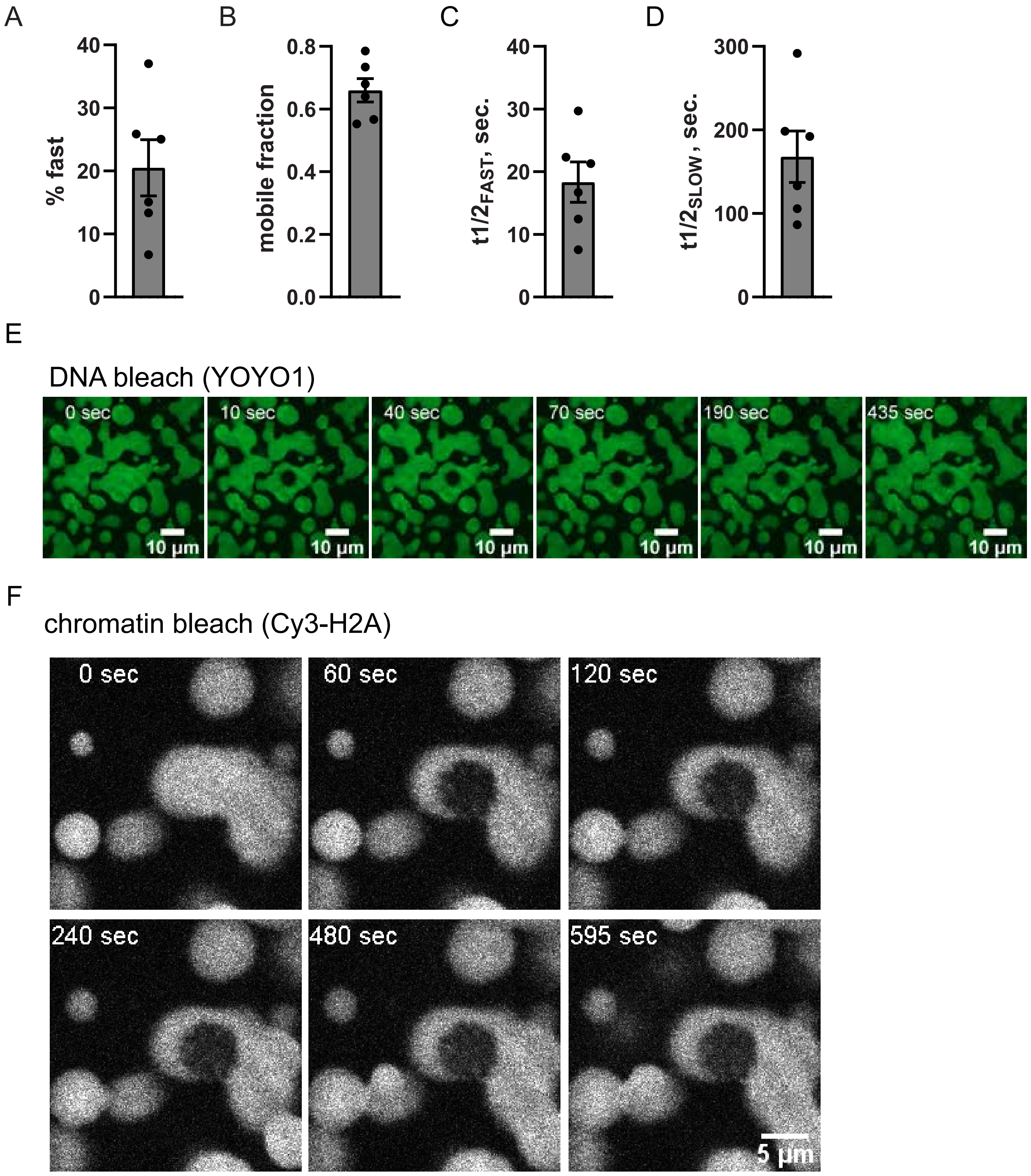

A
B
C
D
40
30
20
10
0
% fast
0.8
0.6
0.4
0.2
0.0
mobile fraction
$t1/2_{FAST}$, sec.
300
200
100
$t1/2_{SLOW}$, sec.
E
DNA bleach (YOYO1)
0 sec
10 sec
40 sec
70 sec
190 sec
435 sec
10 µm
F
chromatin bleach (Cy3-H2A)
0 sec
60 sec
120 sec
240 sec
480 sec
595 sec
5 µm

Supplementary Figure 7 Ph SAM polymerization increases DNA binding affinity of Mini-Ph. A. Representative filters from filter binding experiments to measure DNA binding affinity. B. EMSA demonstrating that Mini-PhΔFCS does not bind DNA. Plasmid DNA was used for EMSA; using these large substrates at high concentrations (25 ng plasmid per reaction) underestimates Mini-Ph DNA binding affinity. Gels are representative of three independent experiments.

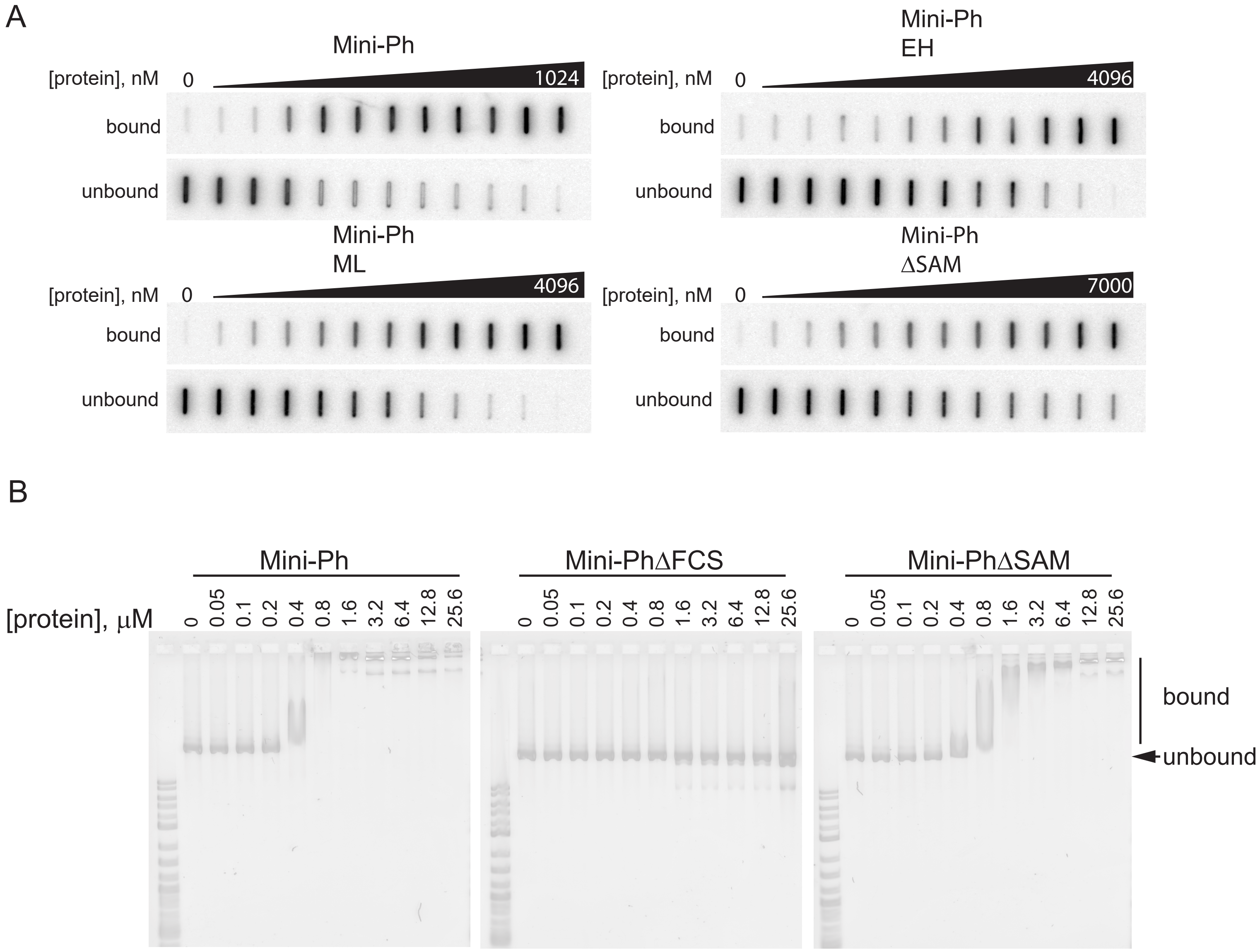
A
Mini-Ph
[protein], nM
0
1024
bound
unbound
Mini-Ph
EH
4096
Mini-Ph
ML
4096
Mini-Ph
ΔSAM
7000
B
Mini-Ph
Mini-PhΔFCS
Mini-PhΔSAM
[protein], μM
0
0.05
0.1
0.2
0.4
0.8
1.6
3.2
6.4
12.8
25.6
bound
unbound

Supplementary Figure 8 Mini-Ph SAM polymerization mutants are more sensitive to NaCl than wild-type. A, B. Matrix of of Mini-Ph ML (A), and Mini-Ph EH (B)-DNA condensates and across different concentrations of NaCl. Images are representative of two independent experiments. C. Plot of one-phase versus two-phase regimens for Mini-Ph, Mini-Ph ML, and Mini-Ph EH. The difference between green and yellow symbols indicates that condensate formation is more sensitive to NaCl for both mutants. See also Supplementary Figure 3 for the matrix of Mini-Ph versus NaCl.

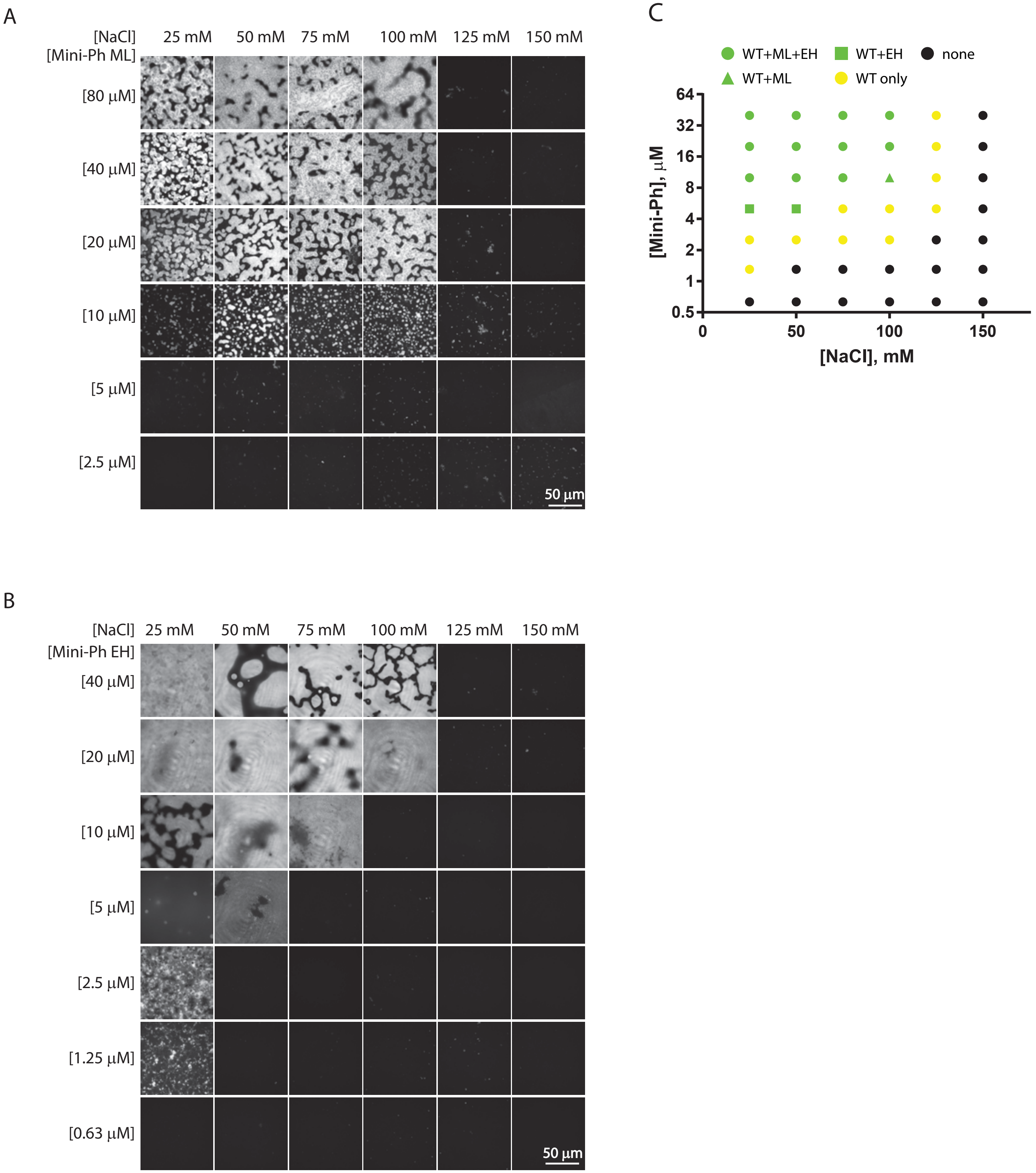

Seif et al., Supplementary Figure 8

Supplementary Figure 9 Mini-Ph SAM polymerization mutants are more sensitive to ATP than wild-type Mini-Ph. A. Schematic of experiments to test effect of adding ATP (or buffer as a control) to condensates formed by Mini-Ph, Mini-Ph ML, or Mini-Ph EH with chromatin. B-E. Representative images (B, D) and quantification (C, E) of condensates after 15 min. incubation with ATP or buffer. n-values are the number of structures measured in this single experiment. F. Effect of 8 mM ATP on condensates formed with Mini-Ph and chromatin. Scale bar in F applies to B, D, and F. The effects of ATP were observed in a second independent experiment with chromatin, and in two experiments with DNA.

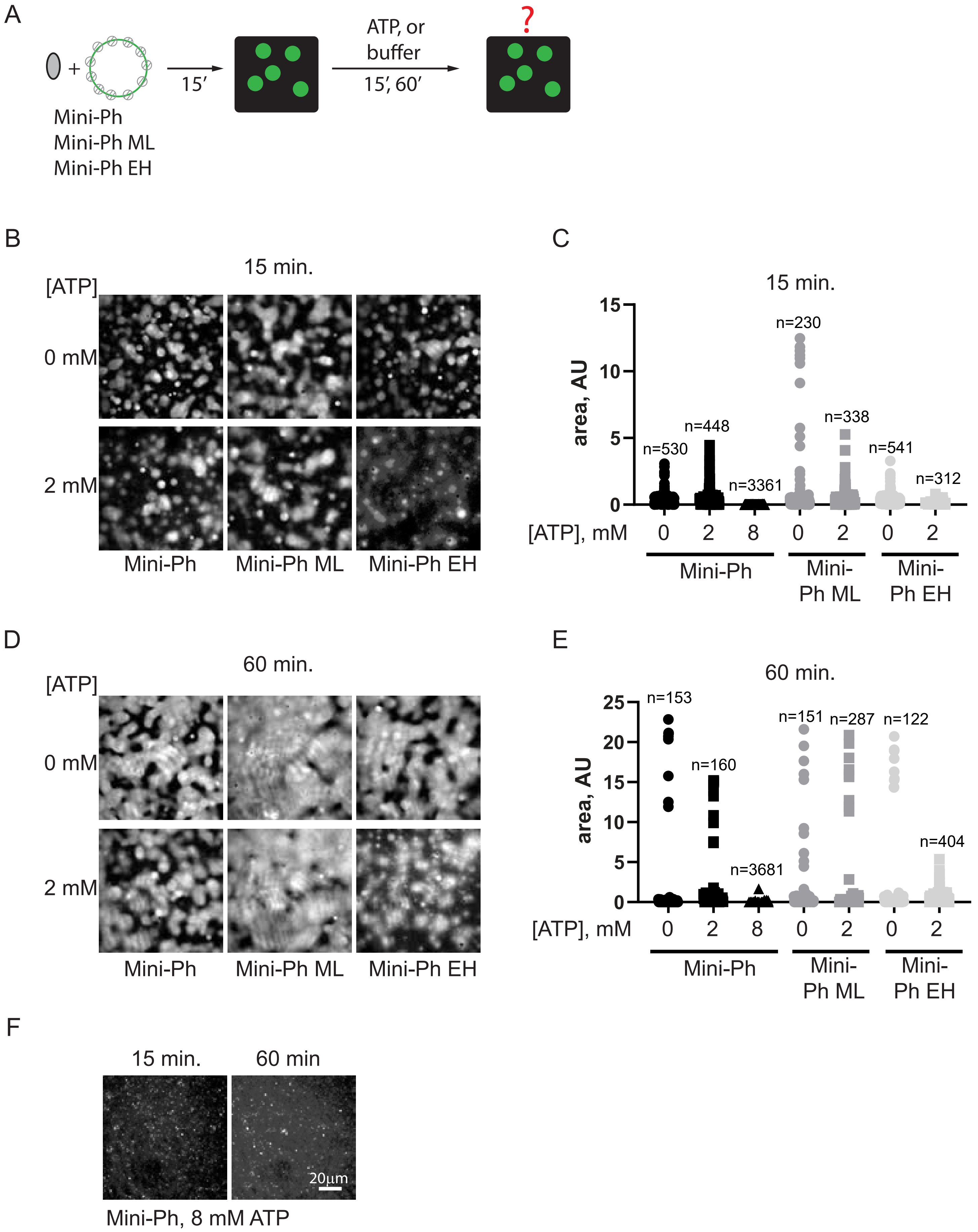
A
Mini-Ph
Mini-Ph ML
Mini-Ph EH
15′
ATP, or
buffer
15′, 60′
?
B
15 min.
[ATP]
0 mM
2 mM
Mini-Ph
Mini-Ph ML
Mini-Ph EH
C
15 min.
area, AU
15
10
5
0
n=530
n=448
n=3361
n=230
n=338
n=541
n=312
[ATP], mM
0
2
8
0
2
0
2
Mini-Ph
Mini-Ph ML
Mini-Ph EH
D
60 min.
[ATP]
0 mM
2 mM
Mini-Ph
Mini-Ph ML
Mini-Ph EH
E
60 min.
area, AU
25
20
15
10
5
0
n=153
n=160
n=3681
n=151
n=287
n=122
n=404
[ATP], mM
0
2
8
0
2
0
2
Mini-Ph
Mini-Ph ML
Mini-Ph EH
F
15 min.
60 min
20μm
Mini-Ph, 8 mM ATP

Supplementary Figure 10 Models for the interactions of Ph SAM in condensate formation. A. Schematic of all known interactions that may occur in Mini-Ph-chromatin or Mini-Ph DNA condensates, including interactions among nucleosomes. B-D. Schematic indicating which interactions are missing when different mutated or truncated proteins that do or do not form condensates are used. The FCS-DNA/chromatin interaction is weaker in the absence of the SAM or when the polymerization interface is disrupted. The residual SAM-SAM interaction in the EH mutant is hypothesized based on the acetylation footprinting results (Supplementary Fig. 13F). The ML mutation, which weakens but does not eliminate SAM-SAM interactions may behave similar to the schematic in A (i.e. the SAM-SAM interaction is dynamic under phase separation conditions, unlike wild-type Mini-Ph which forms short, limited polymers before phase separation). It is important to point out that this is the simplest scheme; it is possible that there are other interactions in the system that have not been characterized. These could include interactions involving the HD1, hinted at by the difference in accessibility measured for Mini-Ph and Mini-Ph EH (Fig. 4D, E). We also do not know how the structure of Mini-Ph polymers influences binding to large chromatin or DNA templates, and whether Mini-Ph binding influences nucleosome-nucleosome interactions (as suggested in the diagrams).

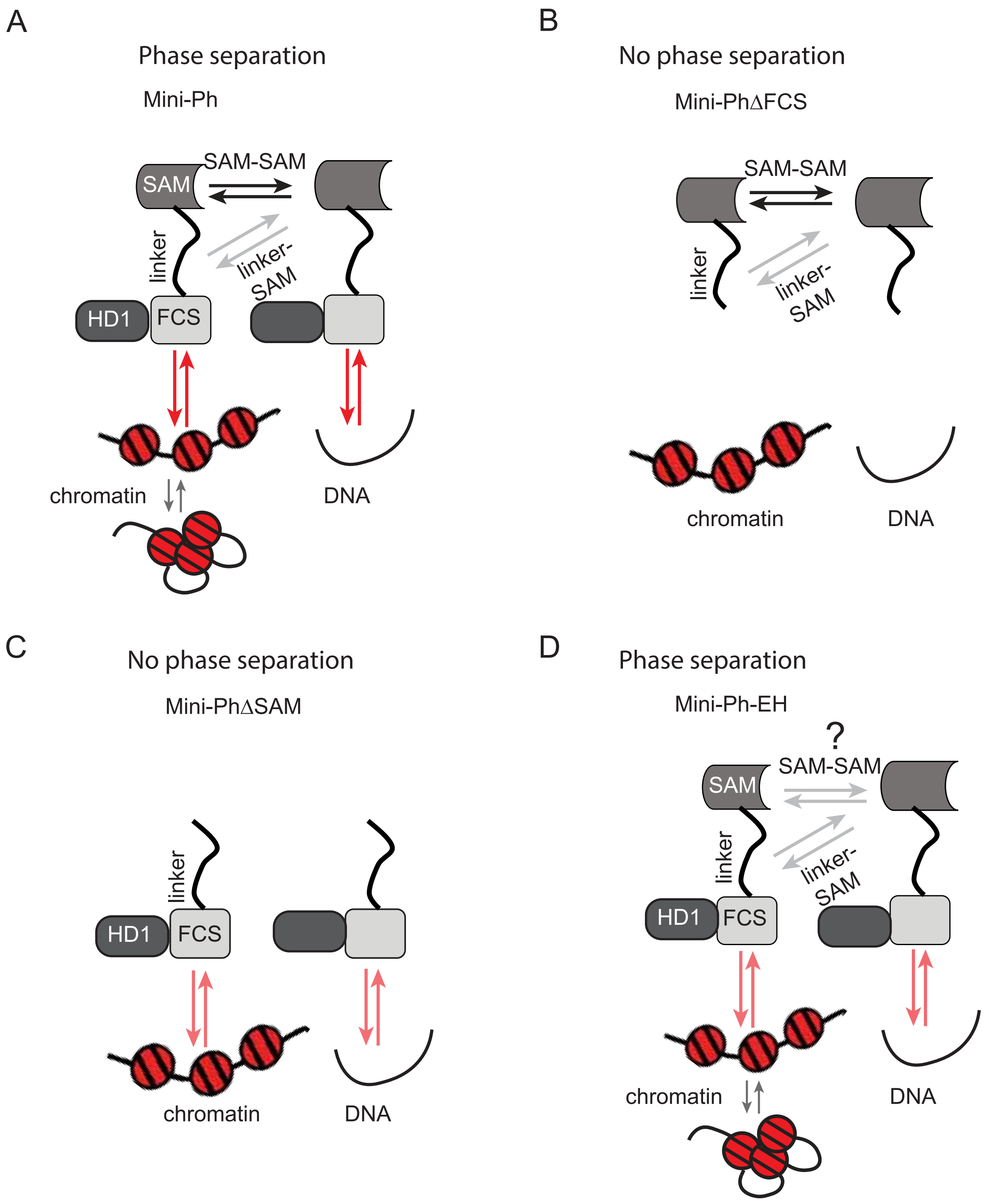

Seif et al., Supplementary Figure 10

Supplementary Figure 11 Chemical footprinting analysis of lysine accessibility of Mini-Ph alone and with DNA. A. Structure of the FCS of human PHC1 (PDB 2L8E), with the positions of the equivalent lysine residues in Ph-p indicated. Red numbers indicate positions where accessibility is significantly changed. B. Clustal alignment of human PHCs with Ph-p indicating the relationship between lysines in Ph-p and the sequence of the FCS. C. Average accessibility of lysines in each Mini-Ph region compared across conditions. Accessibility of all residues in each region was averaged for each replicate and the averages compared across conditions by two-sided student't t-test with Holm-Sidak correction for multiple comparisons. Bars show the mean +/- SEM. n=6.

A

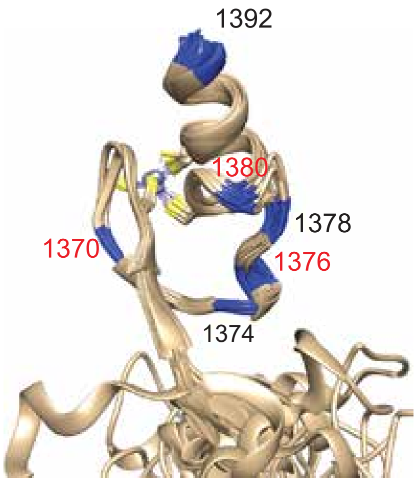

FCS (PHC1)

B

```
                        1376
                    1374 | 1380
               1370     |1378   |          1392
                    |    |  |  |   |          |
Ph_p  1361  DMVACEQCGKMEHKAKLK-RKRYCSPGCSRQAKNGIGGV 1398
PHC1  796   NLLKCEYCGKYAPAEQFRGSKRFCSMTCAKRYNVSCSHQ 834
PHC3  780   ELLKCEFCGKMGYANEFLRSKRFCTMSCAKRYNVSCSKK 819
PHC2  638   LKLKCELCGRVDFAYKFKRSKRFCSMACAKRYNVGCTKR 676
```

C

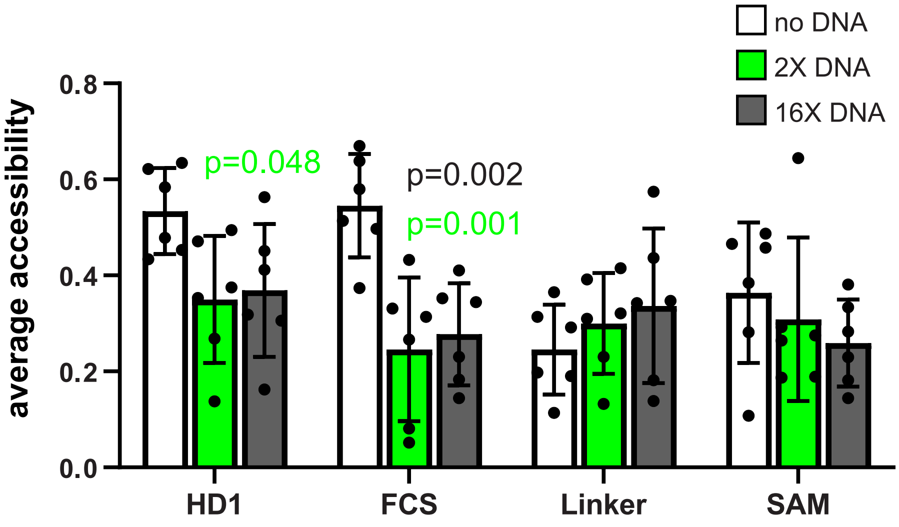

Supplementary Figure 12 Acetylation of Mini-Ph-DNA blocks condensate formation and DNA binding. Time course of effect of sulfo-NHS acetate on Mini-Ph-DNA condensates. Mass spectrometry samples were collected after 15 min. B. Images of high DNA reaction (without acetylation) in which condensates do not form even after prolonged incubation. C. Quantification of time course shown in A. n-values for the number of structures in this single experiment are: control: 517, 555, 564, 352, 146, 98, 80; acetylated: 151, 251, 267, 191, 217, 1306, 4098. A similar time course was observed in three independent experiments. Mean is indicated and bars are SD. D. SYPRO Ruby stained SDS-PAGE of control Mini-Ph and Mini-Ph after acetylation with sulfo-NHS-Acetate. E. EMSA comparing binding of control and acetylated Mini-Ph to DNA; representative of two independent experiments. Main unbound band is supercoiled plasmid, and faint band (grey arrow) is nicked. F. Acetylated Mini-Ph does not form condensates with chromatin (representative of three independent experiments). Images were taken after 60 min. incubation at room temperature.

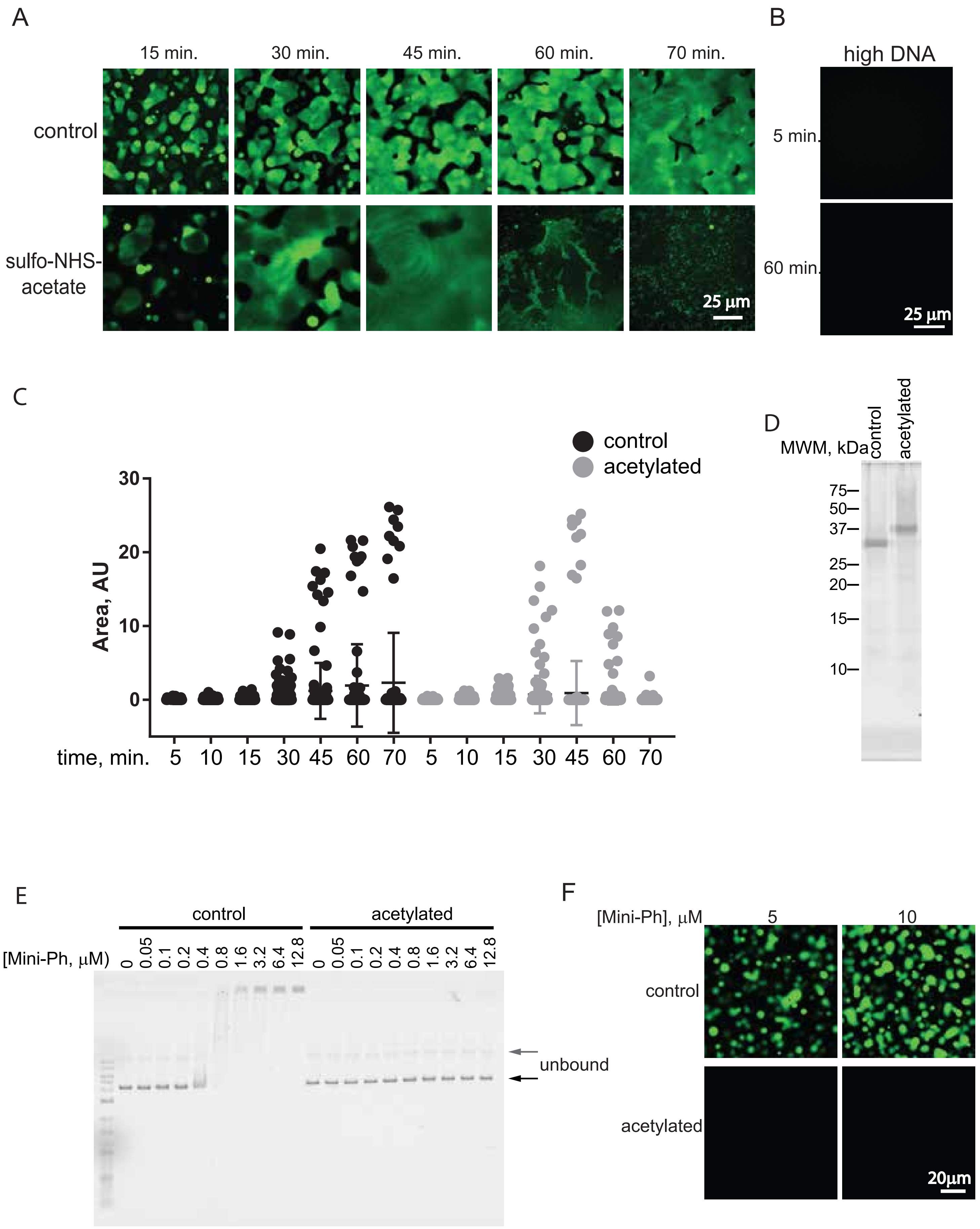

Seif et al., Supplementary Figure 12

Supplementary Figure 13 Chemical footprinting analysis of lysine accessibility of polymerization mutant Mini-Ph EH alone and with DNA. A. Schematic of chemical acetylation (abbreviated from Figure 4A). Acetylation was carried out for 5 or 15 min. with Mini-Ph EH because protein-DNA condensates dissolve rapidly after addition of sulfo-NHS acetate. B. Mini-Ph EH-DNA condensates before (left) and after 5 min. of acetylation (right). C. Heat maps of Mini-Ph EH alone, with 2X DNA (condensates form) or 16X DNA (no condensates). Acetylation was carried out for 5 (n=2) or 15 (n=3) min. Asterisks indicate lysines with significantly different accessibility in the indicated comparisons. Colour scale is from blue (low accessibility) to yellow (high accessibility, from 0-1. D-F. Accessibility averaged over Mini-Ph regions for different conditions after 5 min. (D), 15 min. (E), or 5 versus 15 min. for Mini-Ph EH alone (F). For D-F, n=6 for all conditions for Mini-Ph, and as in C for Mini-Ph EH. Accessibility of all residues in each region was averaged for each replicate and the averages compared across conditions by two-sided student't t-test with Holm-Sidak correction for multiple comparisons. Bars show the error +/- SEM. For n=2 samples (F), no statistics were calculated, and no error bar is shown.

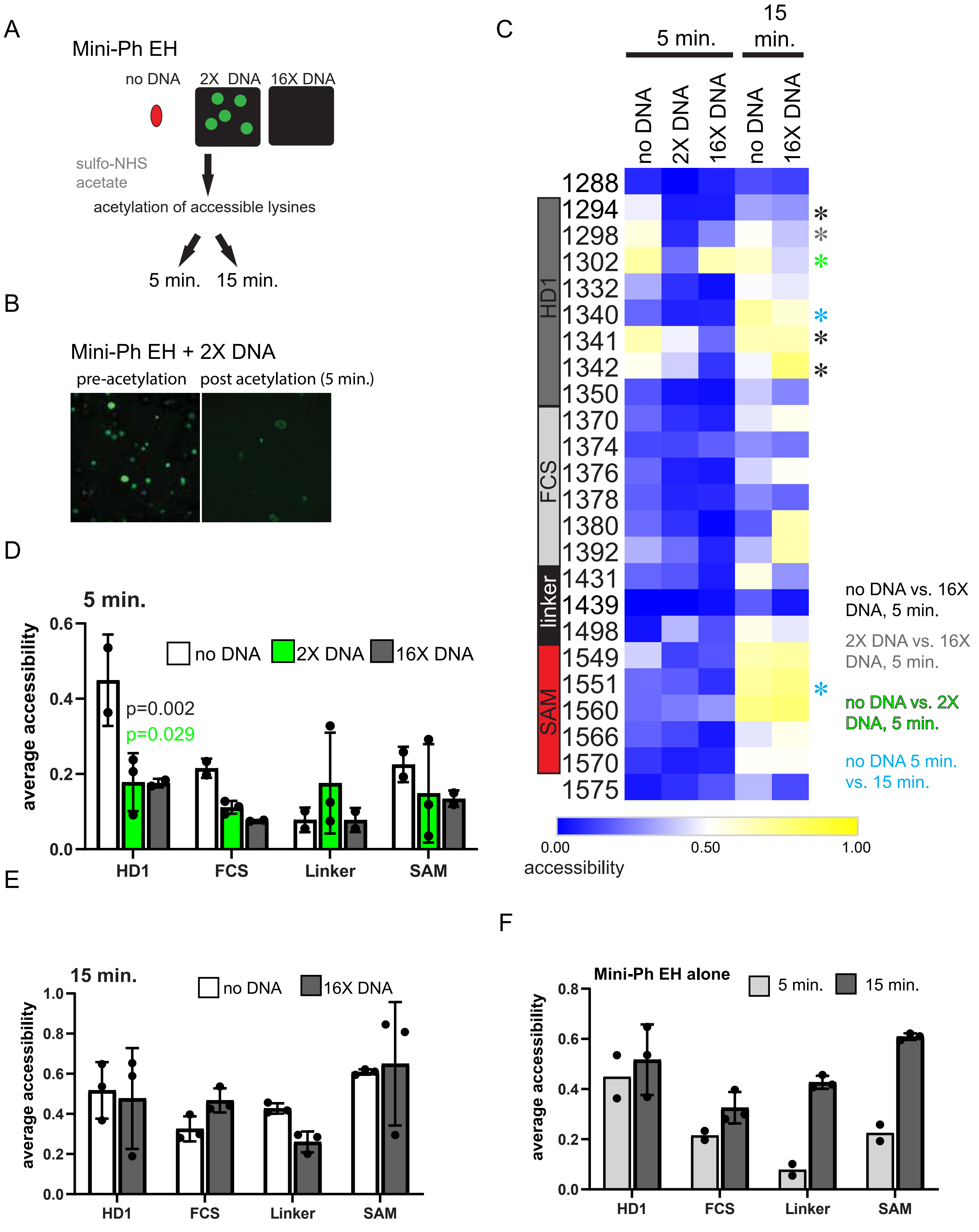

Seif et al., Supplementary Figure 13

Supplementary Figure 14 Mini-Ph EH that lacks polymerization activity is more mobile in condensates than wild-type Mini-Ph. A. FRAP traces for Mini-Ph and Mini-Ph EH with chromatin. Representative traces with R^2 for fits >0.99 were pooled for condensates formed at different times (22-41 min. for Mini-Ph; 28-73 min. for Mini-Ph-EH; n=5 traces for each from two independent experiments). Graph shows the mean +/- SEM. Data were fit with a double exponential equation (Equation 1). B. Fluorescence of chromatin (H2A-Cy3) before and after FRAP experiments. The same regions of interest (ROI) used to measure FRAP, acquisition induced bleaching, and background were used to analyze chromatin as for Mini-Ph from a single experiment. n=9 (WT), n=12 (EH). We were unable to collect FRAP traces in both channels simultaneously. C, D. Representative bleaching and recovery of single condensate for Alexa-647 labelled Mini-Ph (C) and Mini-Ph-EH. Condensates were formed for 25 min. for Mini-Ph and 28 min. for Mini-Ph-EH. E, F. Fluorescence of chromatin (H2A-Cy3) for the same condensates shown in C and D immediately before and after the FRAP experiment. G-I Summary of parameters from double exponential fits. All graphs show the mean +/-SEM. Mini-Ph and Mini-PH EH were compared by Mann-Whitney test; two-tailed p-values are reported. All fits have R^2>0.99. Each point represents a single FRAP trace fit; traces are from three independent experiments.

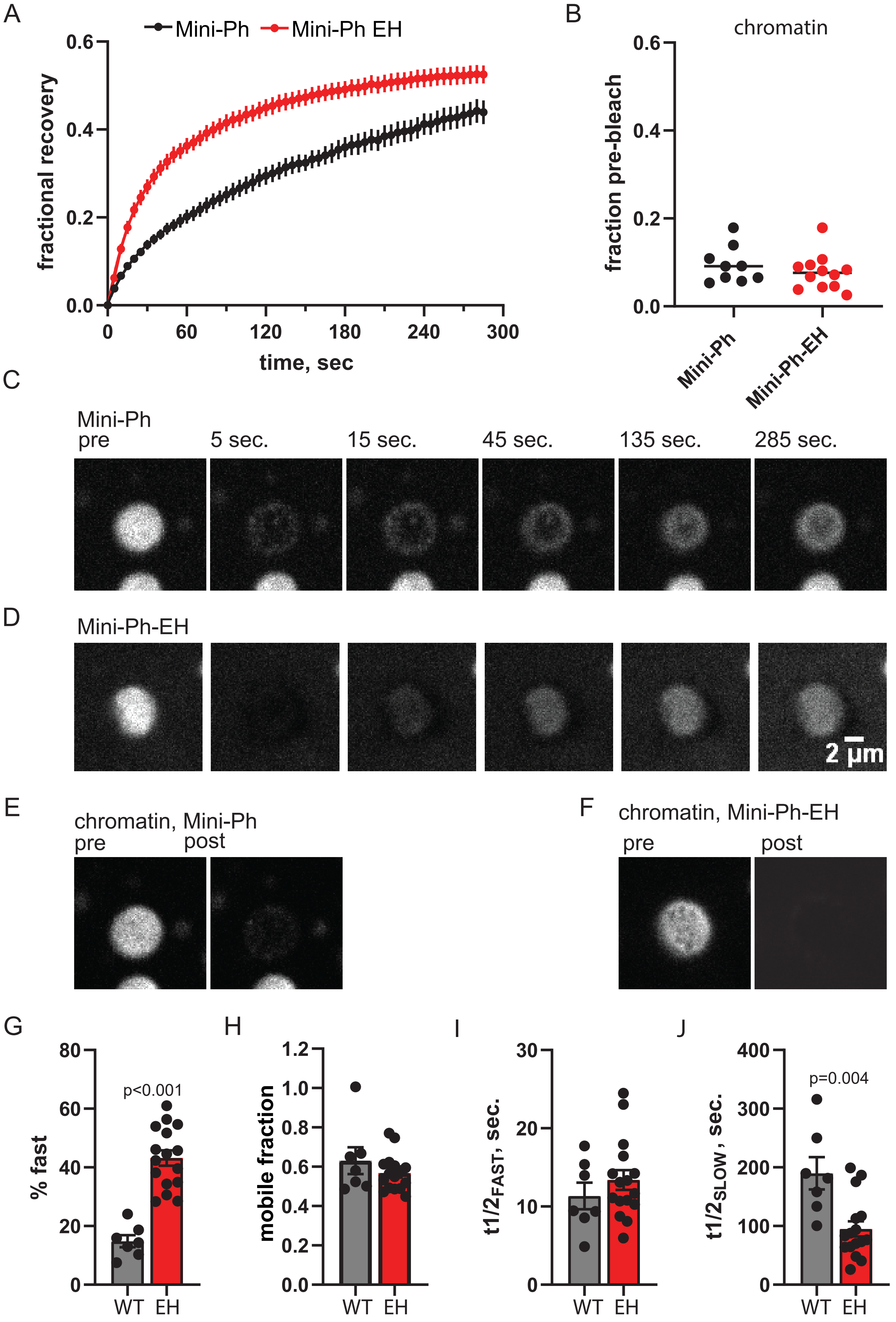
A
Mini-Ph
Mini-Ph EH
fractional recovery
time, sec
0.0
0.2
0.4
0.6
0
60
120
180
240
300
B
chromatin
fraction pre-bleach
0.0
0.2
0.4
0.6
Mini-Ph
Mini-Ph-EH
C
Mini-Ph
pre
5 sec.
15 sec.
45 sec.
135 sec.
285 sec.
D
Mini-Ph-EH
2 µm
E
chromatin, Mini-Ph
pre
post
F
chromatin, Mini-Ph-EH
pre
post
G
% fast
p<0.001
0
20
40
60
80
WT
EH
H
mobile fraction
0.0
0.2
0.4
0.6
0.8
1.0
1.2
WT
EH
I
t1/2FAST, sec.
0
10
20
30
WT
EH
J
t1/2SLOW , sec.
p=0.004
0
100
200
300
400
WT
EH

Supplementary Figure 15 Colocalization analysis of Mini-Ph and chromatin in nuclear extracts. A. SYBR Gold stained gel of nuclear extract (lane 1), extract after desalting (lane 2), and after incubation with Q-sepharose to deplete nucleic acids (lane 3). B. Summary of overlap between Mini-Ph condensates and chromatin incubated in buffer or in nuclear extract from three independent experiments. Bars show the mean, and error bars are SEM. C, D. Representative images from two of the three experiments; two resembled panel C, while in one experiment, Mini-Ph structures that are not positive for Cy3-H2A were observed. These structures frequently appear to be connected to chromatin condensates.

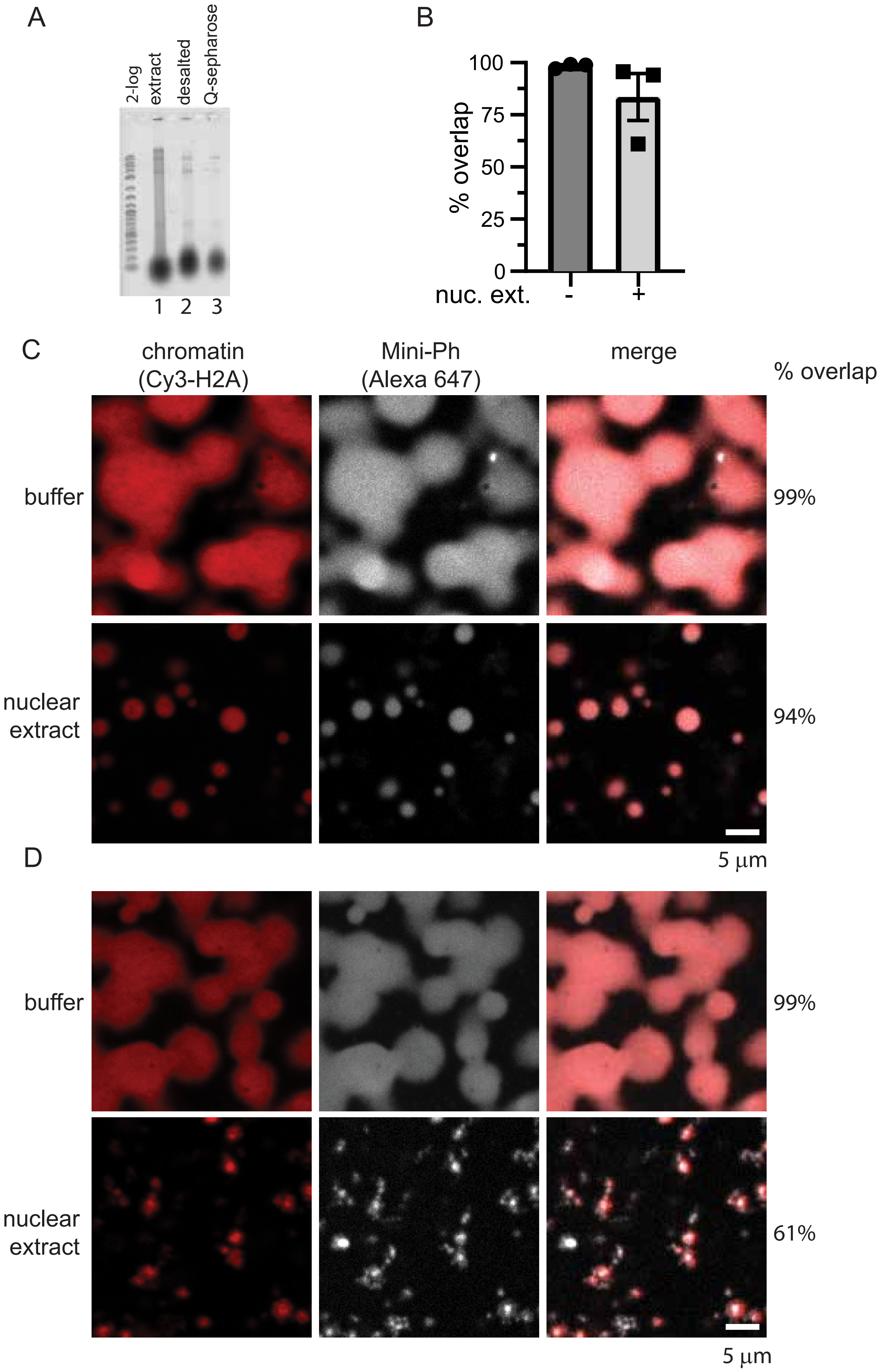
A
2-log
extract
desalted
Q-sepharose
1 2 3
B
100
75
50
25
0
% overlap
nuc. ext.
-
+
C
chromatin (Cy3-H2A)
Mini-Ph (Alexa 647)
merge
% overlap
buffer
99%
nuclear extract
94%
5 μm
D
buffer
99%
nuclear extract
61%
5 μm

Supplementary Figure 16 Effect of nuclear extracts on condensates formed by Mini-Ph, or Mini-Ph polymerization mutants with chromatin. A. Representative images of condensates incubated in buffer (top panel in each set), or nuclear extracts (bottom panel in each set). Image intensities were adjusted to facilitate visualization of the structures. The structures formed by Mini-Ph EH with chromatin in nuclear extract are much fainter than those formed in buffer are. B-D. Average size of structures (B), % area covered by condensates (C), or mean intensity of structures (D) after incubation in buffer or extract from three independent experiments. Bars are the mean +/-SEM and p-values are for one-way ANOVA comparing buffer to nuclear extract for each protein with Sidak correction for multiple comparisons.

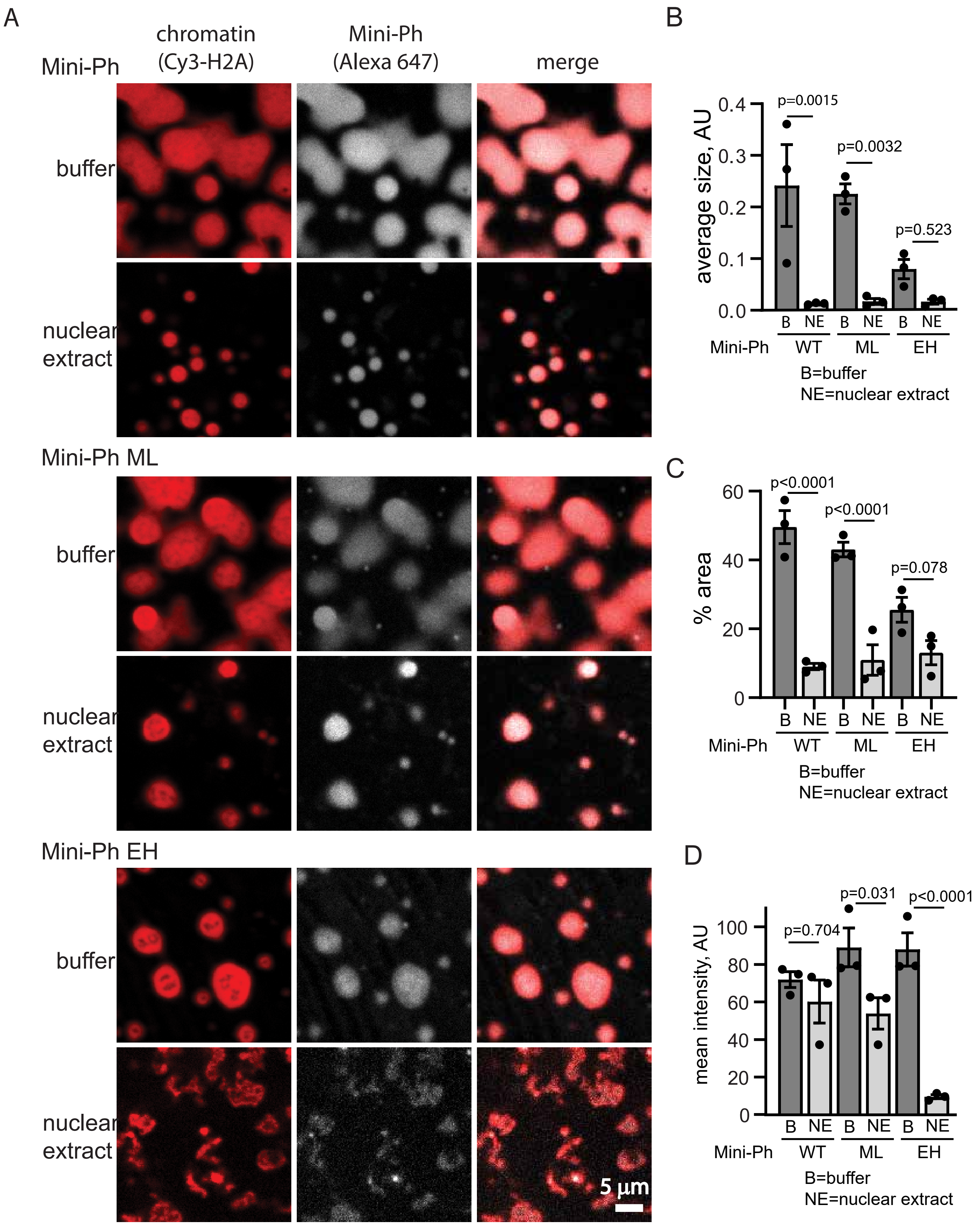

Seif et al., Supplementary Figure 16

Supplementary Figure 17 Histone ubiquitylation assay. A, B. SDS-PAGE of histone ubiquitylation assay. A shows Cy3-labelled histone H2A and B is same gel after SYPRO-Ruby staining. The E1 and E2, ATP, Ubiquitin, and PRC1ΔPh are all required for ubiquitylation. *contaminants present in one gel lane. C. SYPRO Ruby stain of gels shown in Figure 6.

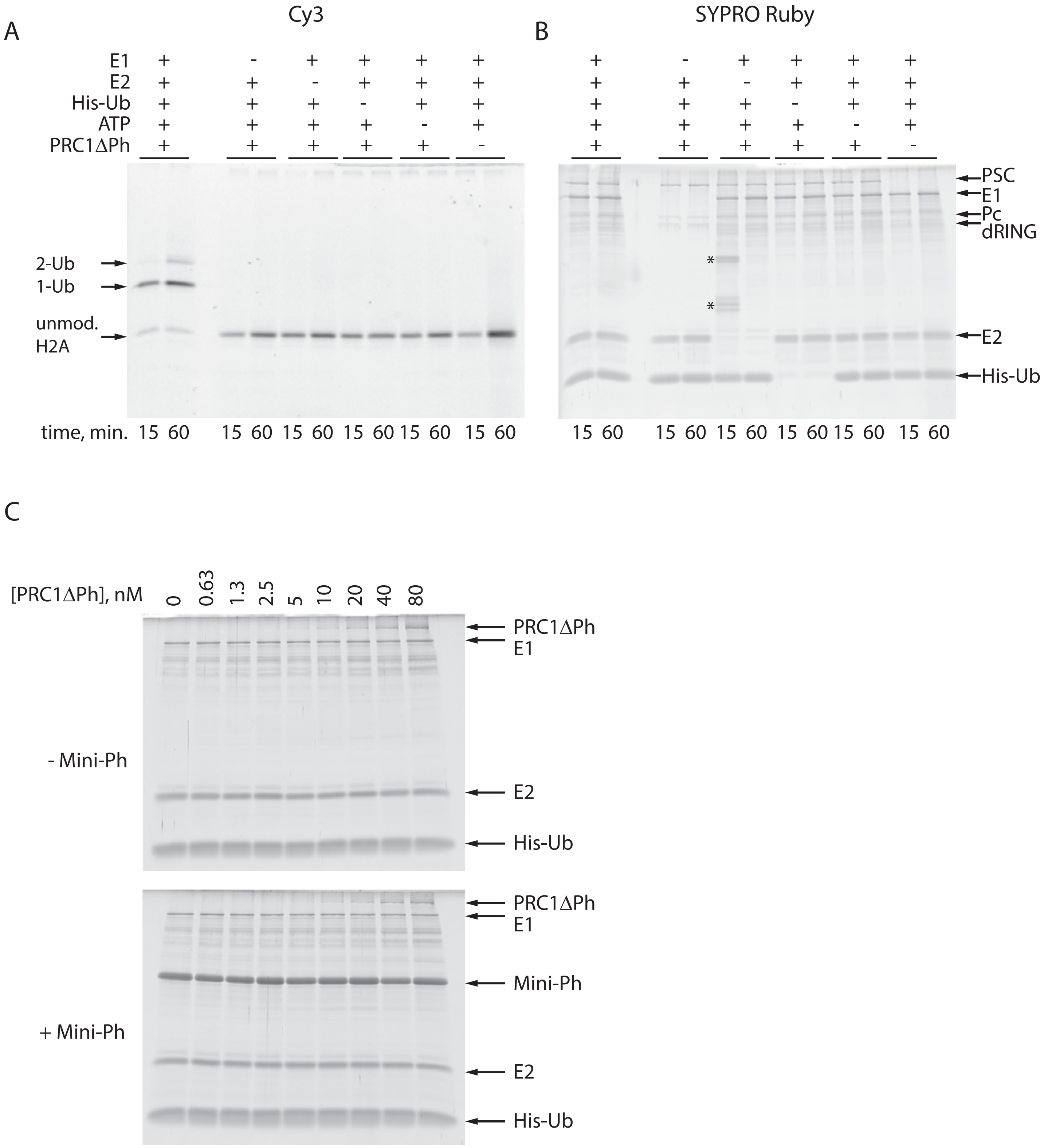

Seif et al., Supplementary Figure 17

Supplementary Figure 18 Mini-Ph forms a single, SAM-dependent focus or no foci in cells. A-C. Representative images of S2R+ cells transfected with Venus-Mini-Ph (A, B) or Venus-Mini-PhΔSAM (C) and H2A-RFP. Cells expressing Venus-Mini-Ph either have a single focus, as in A, or no foci, as in B. We do not observe multiple Mini-Ph foci in cells. D. Quantification of cells with foci. 7% of Venus-Mini-Ph (39/570) and 0.5% of Venus-Mini-PhΔSAM (4/730) cells measured in a single experiment were identified as having a single maxima (focus). Similar results were observed in a second experiment, and Venus-Mini-Ph forms single foci when expressed at higher levels using the UAS promoter and a tubulin driver. In the case of Mini-PhΔSAM, the small number of identified foci represent cells with very high expression rather than actual foci.

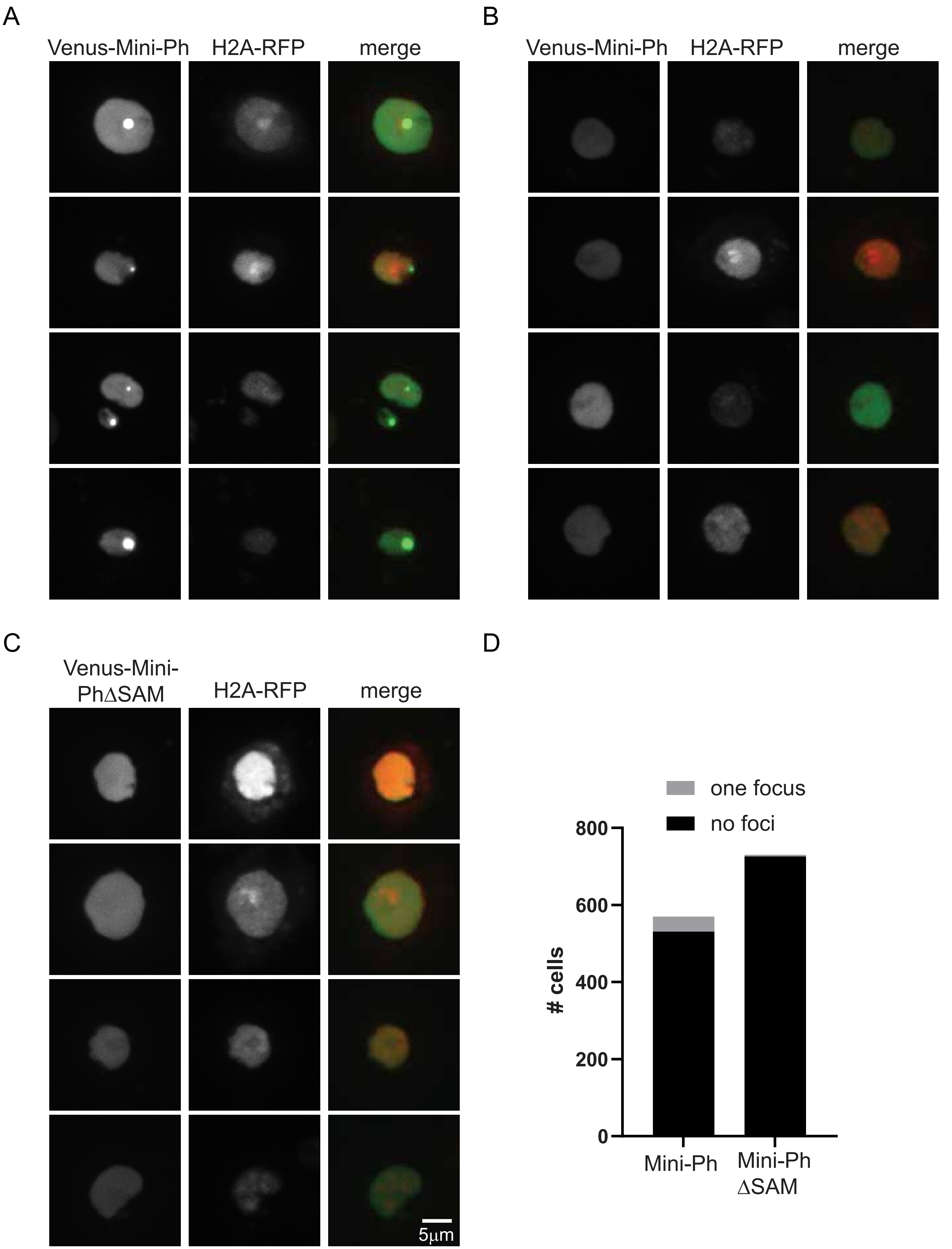
A
Venus-Mini-Ph
H2A-RFP
merge
B
Venus-Mini-Ph
H2A-RFP
merge
C
Venus-Mini-PhΔSAM
H2A-RFP
merge
5μm
D
one focus
no foci
cells
0
200
400
600
800
Mini-Ph
Mini-Ph ΔSAM
