# Supplementary material for: Phase separation by the polyhomeotic sterile alpha motif compartmentalizes Polycomb Group proteins and enhances their activity
Source: Nat Commun. 2020 Nov 5;11:5609. doi: 10.1038/s41467-020-19435-z (PMC7644731; doi:10.1038/s41467-020-19435-z)
Supplement: Supplementary file 3 — Description of Additional Supplementary Files [file 41467_2020_19435_MOESM3_ESM.pdf]

Description of Additional Supplementary Files

File Name: Supplementary Data 1
Description: Analysis of sequence properties of Ph linkers. Table of linker sequences and parameters from localCIDER analysis.

File Name: Supplementary Movie 1
Description: Movie of Fusion of Mini-Ph-Chromatin condensates. Mini-Ph is labelled with Alexa 647.

File Name: Supplementary Movie 2
Description: Movie of Fusion of Mini-Ph-Chromatin condensates. Mini-Ph is labelled with Alexa 647.

File Name: Supplementary Movie 3
Description: Movie of Fusion of Mini-Ph-DNA condensates. Movie shows Mini-Ph labelled with Alexa 647.
