# Supplementary material for: Phase separation by the polyhomeotic sterile alpha motif compartmentalizes Polycomb Group proteins and enhances their activity
Source: Nat Commun. 2020 Nov 5;11:5609. doi: 10.1038/s41467-020-19435-z (PMC7644731; doi:10.1038/s41467-020-19435-z)
Supplement: Supplementary file 8 — Reporting Summary [file 41467_2020_19435_MOESM8_ESM.pdf]

nature research

Corresponding author(s): Nicole J. Francis

Last updated by author(s): Sep 13, 2020

Reporting Summary

Nature Research wishes to improve the reproducibility of the work that we publish. This form provides structure for consistency and transparency in reporting. For further information on Nature Research policies, see our Editorial Policies and the Editorial Policy Checklist.

Statistics

For all statistical analyses, confirm that the following items are present in the figure legend, table legend, main text, or Methods section.

n/a	Confirmed	
☐	☒	The exact sample size (*n*) for each experimental group/condition, given as a discrete number and unit of measurement
☐	☒	A statement on whether measurements were taken from distinct samples or whether the same sample was measured repeatedly
☐	☒	The statistical test(s) used AND whether they are one- or two-sided<br>*Only common tests should be described solely by name; describe more complex techniques in the Methods section.*
☒	☐	A description of all covariates tested
☐	☒	A description of any assumptions or corrections, such as tests of normality and adjustment for multiple comparisons
☐	☒	A full description of the statistical parameters including central tendency (e.g. means) or other basic estimates (e.g. regression coefficient) AND variation (e.g. standard deviation) or associated estimates of uncertainty (e.g. confidence intervals)
☐	☒	For null hypothesis testing, the test statistic (e.g. *F*, *t*, *r*) with confidence intervals, effect sizes, degrees of freedom and *P* value noted<br>*Give P values as exact values whenever suitable.*
☒	☐	For Bayesian analysis, information on the choice of priors and Markov chain Monte Carlo settings
☒	☐	For hierarchical and complex designs, identification of the appropriate level for tests and full reporting of outcomes
☒	☐	Estimates of effect sizes (e.g. Cohen's *d*, Pearson's *r*), indicating how they were calculated

Our web collection on statistics for biologists contains articles on many of the points above.

Software and code

Policy information about availability of computer code

Data collection	ZenBlue (collection of confocal microscopy images)
Data analysis	ImageJ Fiji (image analysis, including FRAP, Western blot quantification), ImageQuantTL (Ubiquitylation assay quantification, Western blot quantification), MaxQuant v1.6.10.43 (acetylation footprinting analysis), GraphPad Prism v8.4.3 (all graphs and statistics), Excel, Morpheus (heat maps)

For manuscripts utilizing custom algorithms or software that are central to the research but not yet described in published literature, software must be made available to editors and reviewers. We strongly encourage code deposition in a community repository (e.g. GitHub). See the Nature Research guidelines for submitting code & software for further information.

Data

Policy information about availability of data

All manuscripts must include a data availability statement. This statement should provide the following information, where applicable:

- Accession codes, unique identifiers, or web links for publicly available datasets
- A list of figures that have associated raw data
- A description of any restrictions on data availability

Mass spectrometry raw files will be uploaded to are available at MassIVE (ftp://massive.ucsd.edu/MSV000085717/). Source data are provided with this paper. The Source Data file includes data for FRAP traces and fits (Fig. 2, Supplementary Fig. 146, 14), and filter binding data (Fig. 3C), nucleosome and condensate measurements (Fig. 3H, I, J), MaxQuant output (intensities) for acetylation footprinting experiments (Fig. 4), filter binding data (Fig. 3C), nucleosome and condensate measurements (Fig. 3H, I, J), western blots and quantification (Fig. 5I, 7C, DFig. 5, 7), ubiquitylation activity assay quantification (Fig. 6E), foci measurements (Fig. 7H). All other raw data are available on reasonable request.

Field-specific reporting

Please select the one below that is the best fit for your research. If you are not sure, read the appropriate sections before making your selection.

☒ Life sciences ☐ Behavioural & social sciences ☐ Ecological, evolutionary & environmental sciences

For a reference copy of the document with all sections, see nature.com/documents/nr-reporting-summary-flat.pdf

Life sciences study design

All studies must disclose on these points even when the disclosure is negative.

Sample size	Biochemical experiments were typically conducted at least three times (independently), and with two different chromatin preparations. In cases where a smaller sample size is used, it is because a similar but non-identical experiment produced the same result.
Data exclusions	For FRAP data, traces that could not be fit with confidence or had obvious technical artifacts were excluded. For filter binding data, a small number of points that are clear technical artifacts were excluded. These are indicated in the source data file.
Replication	Experiments were conducted multiple times (as described above). For biochemical experiments we have typically also done multiple versions of each type of experiment (for example different incubation times or more extensive titrations or conditions than presented).
Randomization	not relevant to this study
Blinding	not relevant to this study

Reporting for specific materials, systems and methods

We require information from authors about some types of materials, experimental systems and methods used in many studies. Here, indicate whether each material, system or method listed is relevant to your study. If you are not sure if a list item applies to your research, read the appropriate section before selecting a response.

Materials & experimental systems

n/a	Involved in the study	
☐	☒	Antibodies
☐	☒	Eukaryotic cell lines
☒	☐	Palaeontology and archaeology
☒	☐	Animals and other organisms
☒	☐	Human research participants
☒	☐	Clinical data
☒	☐	Dual use research of concern

Methods

n/a	Involved in the study	
☒	☐	ChIP-seq
☒	☐	Flow cytometry
☒	☐	MRI-based neuroimaging

Antibodies

Antibodies used	Primary antibodies are as follow: anti-Ph (Rb) (prepared in Francis lab) 1:2000; anti-Pc (Rb) & anti-Su(Z)12 (Rb((gifts of J. Mueller) 1:5000, 1:3000; anti-Acf1 (Rb) (gift of D. Fyodorov), 1:1000; anti-RPA (Rb) (gift of P. Fisher), 1:3000; anti-p55(Abcam), 1:1000.
Validation	Antibodies used react with proteins at the expected size. Several of the antibodies used here (i.e. against PcG proteins) have also been validated with recombinant protein, and using RNAi against the target protein (Ph, Pc, Su(Z)12, PSC).

Eukaryotic cell lines

Policy information about cell lines

Cell line source(s)	Drosophila 52 Cells, Expression Systems and Drosophila 52R+ cells, Drosophila Genome Research Center
Authentication	N/R
Mycoplasma contamination	mycoplasma contamination was tested (PCR test) and not observed
Commonly misidentified lines (See ICLAC register)	N/R
